# Preloading with drugs before entry to the nighttime entertainment district: presentation, intoxication rates, and effects of police presence during assessment

**DOI:** 10.1186/s12954-023-00749-2

**Published:** 2023-05-02

**Authors:** Lee R. J. Hughes, Corey Allen, Grant J. Devilly

**Affiliations:** 1 School of Applied Psychology, Griffith University, Mt Gravatt, QLD 4122 Australia; 2 Griffith Criminology Institute, Mt Gravatt, QLD Australia; 3 Queensland Police Service Academy, Queensland Police Service, Brisbane, QLD Australia

**Keywords:** Illicit drugs, Alcohol use, Preloading, Police presence, Field research, Nighttime entertainment districts

## Abstract

**Background:**

Past research has either focused on alcohol or drug preloading before a night out, but not on the interaction between them. With increased risks of harm through interaction effects, we wished to build upon previous research in this area. We sought to determine who drug preloads, why do people engage in this practice, what drug/s are people using, and how inebriated they are as they enter the NED. Additionally, we examined what impact varying levels of police presence has on the collections of sensitive data in this context.

**Methods:**

We captured estimates of drug and alcohol preloading from 4723 people entering nighttime entertainment districts (NEDs) in Queensland, Australia. Data collection occurred under three varying conditions of police presence (i.e., no police present, police present but not engaging with participants, and police engaging with participants).

**Results:**

People who admitted to preloading drugs were found to be younger in age than non-drug admitters, more likely to be male than female, use one type of drug (mostly stimulants) rather than multiple (if we exclude alcohol), significantly more intoxicated upon arrival, and more subjectively affected from their use of alcohol and drugs as Breath Approximated Alcohol Concertation levels increased. People were more likely to admit having used drugs in the absence of police, but this had only a small effect.

**Conclusions:**

People who drug preload are a vulnerable subset of the youth population that is susceptible to experiencing harms in this context. As they drink more alcohol, they experience higher affects than those who do not report to also take drugs. Police engagement through service rather than force may mitigate some risks. Further enquiry is needed to better understand those who engage in this practice and to have quick, cheap, objective tests of what drugs these people are using.

**Supplementary Information:**

The online version contains supplementary material available at 10.1186/s12954-023-00749-2.

## Background

The act of preloading (also referred to as pre-gaming, pre-partying, and front-loading) has become a prevalent start to nights out for many partygoers venturing into nighttime entertainment districts (NEDs; [3]. To date, most investigations into preloading have focused specifically on alcohol use only, whereas the use of drugs^1^ during this phase of the session has seldom been explored [20]. Consistent with this, past research has either focused on alcohol preloading or drug use, and not on the interaction between them. This warrants concern because findings from epidemiological research suggest that people preload with alcohol and drugs before transitioning into nightclubs and bars [14, 15, 28, 29], where it is common for them to continue using substances over the course of the night [28, 29, 32, 33, 35]. In the present study, we seek to bridge these gaps in the literature by shedding much needed light on the nature of drug (and polysubstance) preloading. To achieve this, we seek to determine: who preloads with drugs, why people preload with drugs; what drugs people preload with; and what impact does this have on how inebriated they are upon entering the NED. Considering the paucity of research on the topic of drug preloading, we also seek to further contribute to discussions surrounding the safe and ethical collection of sensitive data in field research.

For well over a decade preloading has steadily gained notoriety among research academics, police and emergency services, policy makers, and media outlets due to the risks and dangers associated with this practice [2, 18]. Although the literature base has grown expediently over the course of this time to further our knowledge of preloading, so too has the scope of this practice, with new and emerging trends becoming more salient [20]. In a recent analysis of preloading, Hughes and Devilly [20] evaluated and discussed the importance of operationalizing the parameters with which the construct is being explored. The absence of this could lead to important factors being overlooked (e.g., the disconnect between alcohol preloading and drug use) and methodological problems (e.g., errors associated with measurement and/or sample representation), both of which negatively impact the validity of conclusions in a study. This prompted the authors to develop of a criterion-based model of preloading that emphasizes sensitivity and specificity to mitigate this concern. In line with this approach, we define preloading as the use of alcohol and/or other substances, either individually or in groups, at a private residence or closed space (e.g., at home, a friend’s house, or hotel/motel/hostel) and/or public space (e.g., a park, on public transport, suburban sports club/bar/pub), before going to a target-event (i.e., licensed night clubs and pubs located inside NEDs [20]). Considering the myriad of freedoms these social settings provide (i.e., weakened or absence of constraints found in NED venues), it is not surprising that people are engaging in riskier behaviors (e.g., rapidly consuming excessive amounts of alcohol during a drinking game or mixing alcohol with drugs [10, 12, 38, 44]) and are presenting to NEDs highly intoxicated [13-15, 21].

Risky practices such as preloading are often sensitive to environmental factors (e.g., tax, cost, cigarette smoking practices, accessibility of substances, and increased rules and regulations inside licensed venues), and with change comes new challenges for researchers and other key stakeholder groups to overcome. For instance, prior to the implementation of legislative^2^ change in Queensland, Australia, it was unclear whether strict controls at licensed premises and changes to lockout times would merely encourage people to increase their preloading of alcohol and/or seek alternative methods to achieve their desired level of intoxication for the evening. Findings from field research that examined the before-to-after effects of these legislative reforms revealed that there was a growing subculture of people who preload with alcohol and drugs before entering NEDs. Additionally, it was reported that people were preloading more frequently, were entering the NED later, and were significantly more inebriated upon arrival [14, 15]. Those findings coincided with other research that suggests people are requiring more resource intensive aid earlier in the night [16]. Considering the risky behaviors associated with alcohol preloading and drug use (at any point during the session), it is evident that there is a need to better understand the full scope of this practice.

In our earlier synthesis and reviews of the preloading literature, we sought to determine who preloads with drugs and better understand the nature of the event itself. Drawing from the seminal works of Devilly and colleagues (Australia), Miller, Voas, and colleagues (North America), and Sanchez and colleagues (South America), we could identify that there is a small subset of people who preload with drugs in this context. Besides this, we were unable to identify anything else about drug preloaders without also encountering various methodological and ethical issues. In turn, much of what could be deduced from people who drug preload before entering NEDs remains speculative and should be interpreted with caution. Some key themes we identified included:

- There were no consistent identifiable themes regarding the personal characteristics of drug users other than they tended to be male, heterosexual, and employed full-time.
- Cannabis had the highest incidence of use. This was followed by cocaine and other stimulant type drugs (i.e., 3,4-methylenedioxymethamphetamine [MDMA], amphetamines, and methamphetamine). The use of all other substances was far less prevalent.
- Drug use was often associated with engagement in high-risk behaviors such as polysubstance use (most drug users in these studies were revealed to be polysubstance users), engagement in heavy episode drinking, and driving while intoxicated.

The act of alcohol preloading has long been regarded as an integral part of the evening that is given just as much, if not more, thought than the latter phases of the evening [3]. However, it is yet to be determined why people preload with drugs and whether people do so for the same reasons as people who preload with alcohol only. As it currently stands, the reasons people alcohol preload have shown to greatly vary and are often driven by individual circumstances (e.g., to save money, [26, 28, 29, 37]), desired outcomes (e.g., to socialize with friends, [13, 26]), and social influences and situational contexts (e.g., peer pressure or to enhance safety and have control over the environment, [3, 13, 25, 34, 41]). To the best of our knowledge, it is yet to be determined if people preload with drugs in the same manner, and for the same reasons, that they preload with alcohol. Consistent with alcohol preloading, a similar pattern of behaviors was reported by Coomber et al. [7] in their investigation into the social supply of drugs. Qualitative reports in this study suggest that access to drugs is often organized by designated buyers, based on a preference by individual drug users to prevent precarious interactions with drug-dealers, while also representing a reliable, convenient, and economical option for obtaining drugs. In essence, the preloading environment provides a viable and practical setting for social supply to occur given its shared themes with alcohol preloading (i.e., a valued social aspect, an increased desire for safety, and reduced financial expenditure). Moreover, some people might preload with drugs as a practical approach to avoid detection and persecution from the police and/or venue security [28, 29].

**Fig. 1:**
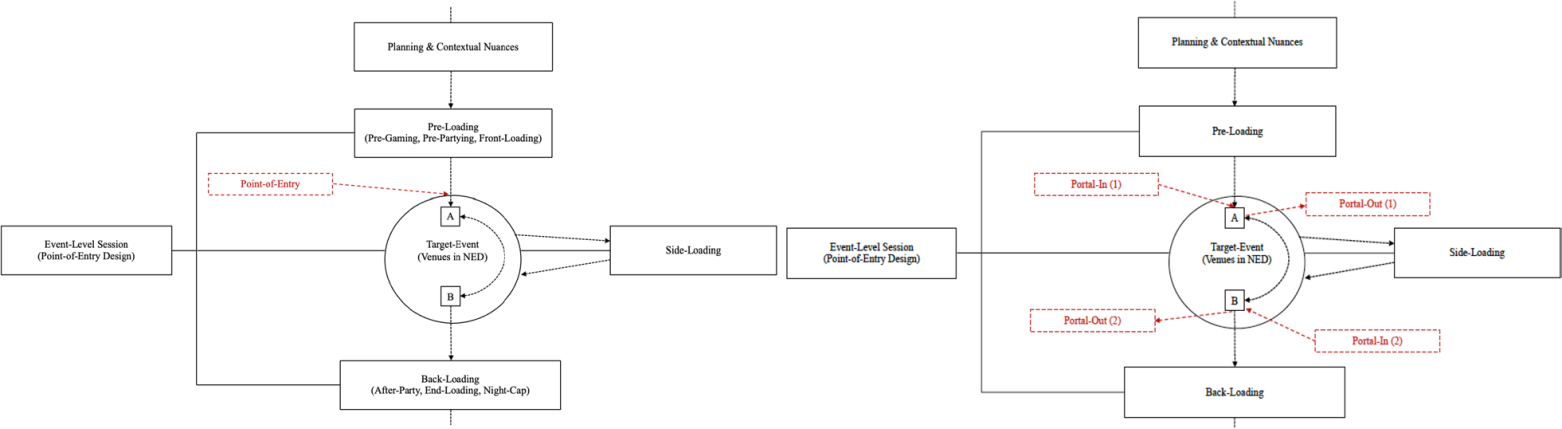
A visual representation of the point-of-entry (left) and portal-design (right) methodologies as applied to the broader event-level session. Note: The red arrows mark the point of intercept, whereby people are recruited to participate in the study. The phases (i.e., preloading, target-event, side-loading, and back-loading) of the event-level session are all interconnected by the straight lines. The dotted line signifies the trajectory a person makes as they transition through the phases of the session. The squares labeled ‘A’ and ‘B’ inside the target-event represent venues inside the NED. The doubled arrow connecting venues ‘A’ and ‘B’ denote a person’s ability to move between venues

Through our review of the preloading literature, we identified that two suitable field-based methodologies researchers have used to report on drug use and preloading behaviors. These approaches include: (1) recruiting participants as they portal-in (entering component of the portal-design) to nightclubs and bars (e.g., [28, 29, 32, 33]) and (2) intercepting people at point of entry into the NED (e.g., [13, 14, 14, 15, 15]). Although these approaches share some similarities, both have shown to serve different functions and can offer different levels of sensitivity and specificity depending on how they are applied in the field. As it currently stands from the literature, we consider point-of-entry designs to have greater sensitivity than portal-in designs in preloading research. Our reasoning for this stems from there being greater assurance that people had not yet entered the licensed venues in studies using the point-of-entry design. However, to the best of our knowledge, research using this design has either: (a) not reported on drug use, (b) only reported the incidence rate of people disclosing their use of drugs and nothing else, or (c) collected data in the presence of police. To date, much of what can be gleaned about drug preloading comes from research using the portal-in design. However, from this research it is often unclear whether the participants were transitioning from the preloading phase into the target-event (i.e., they have not yet entered a licensed venue and ingested more of a substance), or, whether they had consumed substances inside other clubs and bars after having already transitioned into the NED. It may be that researchers clarified this with the participants, but this is not always mentioned in study descriptions. Without this distinction, any inferences made about drug preloading are only speculative. To bridge this gap, we seek to obtain information on people who preload with drugs before entering the NED using Devilly et al.’s [13] point-of-entry design. This issue can be observed visually in Fig. 1, whereby we provide a comparison between the point-of-entry design and the portal-in design (mapped on to our model of the event-level session).

### The present study

The current study has both a substantive and methodological aim. Our substantive aim is to assess the personal characteristics of those that preload with drugs; examine what drugs and polysubstance combinations people typically preload with; determine why people preloaded with drugs; and measure markers of intoxication and impairment severity at point of entry into the NED. We assess markers of alcohol intoxication through measures of Breath Approximated Blood Alcohol Concentration (BrAC) severity and subjective ratings of how affected a person feels from their substance use. All data were collected at point of entry into the NED. As we conducted this study over multiple years, our methodological aim is to explore the impact of varying levels of police exposure (i.e., with the police present [prior to the legislation change] and without the police present [after the legislation change]) has on participant recruitment and drug disclosure in field-based research (see Additional file 1 for a more detailed discussion on our ethical considerations for this research). To explore this further in the present study, we will create another condition and include participants who actively engage with the police chaperone. We start by first addressing the methodological aim as a manipulation check given the impact it could have on our results.

### Exploratory hypotheses

#### H1

It is hypothesized that a subset of the population will preload with drugs, but that:

- People will be least likely to admit to drug preloading when there is a police presence (with no proactive engagement from the officers) around researchers;
- More likely to admit to drug preloading after police have engaged with participants in a positive manner and reinforced that this is research and not a ‘sting’; and
- Most likely to admit to drug preloading when there are no police present.

#### H2

Looking at those *‘who’* drug preload, it is expected that:

- They will be younger in age than non-drug users; and
- Males will be more likely to engage in this practice than females.

#### H3

Looking at ‘*why*’ people preload with drugs, it is hypothesized that:

- Peoples’ primary motivation to do so will be socially driven.

#### H4

Looking at ‘*what*’ drugs people preload with, it is expected that:

- We will see a diverse pattern of recreational drug types and use at preloading; and
- There will be more single-drug users than poly-drug users (when excluding alcohol use as a drug).

#### H5

Looking at ‘*how*’ people preload with drugs, it is hypothesized that:

- more drug users would have also used alcohol than not used alcohol;
- drug users would arrive more intoxicated than non-drug users; and
- will present feeling more affected by their use of substances (including alcohol) than non-drug users.

## Methodology

### Participants and procedure

All study procedures were cleared by the Griffith University Human Research Ethics Committee (PSY/71/14/HREC & 2015/704). This study was completed as part of a larger research project that examined the substance use habits of people entering and exiting NEDs before and after legislative change in Queensland, Australia (cf. [13] for detailed description of our methodological approach). Data collection occurred from August 21, 2014, to September 2, 2017.

A total of 4723 participants were recruited Thursday night to Sunday morning between the hours of 8:50 p.m. to 5:00 a.m. from popular thoroughfares (i.e., main bottlenecks outside major public transport hubs, bars, and clubs) leading into the NED. Researchers approached every fourth person, or group of people, and invited them to participate in the study. Following a refusal, each subsequent passer-by was asked to participate. Although we do not have refusal rates for all nights of our data collection, similar research using this methodology has found a 14.67% refusal rate.

All participants provided verbal assent prior to partaking in the research. Prior to participation, each participant was given a research card containing a unique identification number and contact details should they choose to withdraw their data. All survey data were collected anonymously and away from police officers. Participants completed in situ surveys that were presented to them by the researchers. After completing the survey, participants were administered the BrAC test by the researchers. Once the sample was analyzed, participants were then provided with their exact BrAC reading to three decimal places and an interpretation of their result.

To provide context between testing conditions, officers in the ‘*police presence*’ condition were stationed around the general vicinity of the data collection site. The officers did not actively engage with participants in this condition unless risk was imminent. Officers were stationed in closer proximity to researchers in the ‘*police engagement*’ condition. The role of the police officers in this condition was to greet passers-by, explain what we were doing, assure them it was research, and interpret BrAC readings after a participant had completed both the survey and BrAC test. (Prior to administering the survey all participants were moved from the officers to a location where privacy was ensured.) In the ‘*no police*’ condition, researchers informed venue staff, police, and other relevant street-based community services to make their presence known for the purpose of information, with all participation occurring in absence of these services. Irrespective of testing conditions, researchers have not required any police intervention to ensure safety.

## Materials

All measures were kept brief to encourage participant engagement and reduce malingering. We used QuickTapSurvey [42] loaded onto an iPad/Android tablet to document survey responses and BrAC readings. See Additional file 1 for a detailed description of our survey questions. Participants were breathalyzed using the Alcolizer LE5 (LE5, Alcolizer; Alcolizer Pty Ltd., Brisbane, Queensland, Australia) fuel-cell breathalyzer [1]. The LE5 is the only handheld breathalyzer device to have demonstrated good reliability and validity in NED field trials (cf. [40]).

### Approach to analysis

Survey data were analyzed using SPSS v.28 (IBM [22], St. Leonards, NSW, Australia) and Statistica (TIBCO Software Inc., 2018). All variables were screened for clerical errors, missing values, and assumption violations prior to data analysis. While all *p* values < 0.05 are interpreted as statistically significant, effect sizes were computed to avoid overvaluing a significant result to due to the large sample size [17].

## Results

### Legislative change and police presence

As there was legislative change during our data collection, we first needed to check for differences caused by this introduced variable. For people before the legislative change, police presence was associated with lower reported incidence of admitted drug use than with no police presence, *χ*^2^(*df* = 1, *n* = 3326) = 23.44, *p* < 0.001; *φ* = 0.084. We did not have a police presence during data collection after the legislative change, but there was no significant change in the reported incidence of drug use in the no police condition from before to after the change, *χ*^2^(*df* = 1, *n* = 1952) = 0.71, *p* = 0.40; *φ* = 0.019. Combining both before and after legislative change, and hence increasing power, we find that, as hypothesized (H1a), police presence was associated with lower reported incidence of admitted drug use, *χ*^2^(*df* = 1, *n* = 4314) = 40.02, *p* < 0.001; *φ* = 0.096. In effect, such a large sample size meant that we were sensitive to small differences and the difference between the police presence and no police conditions was very small indeed. (Only 0.92% of the variance is attributable to the relationship between police presence and admitted drug use.)

Unsupportive of hypothesis 1b, the association between the police presence and police engagement conditions was not significant, *χ*^2^(*df* = 1, *n* = 2771) = 2.38, *p* = 0.12; *φ* = − 0.029. However, there was a higher reported incidence of disclosed drug use after police had engaged with the participants compared to when they were just present around testing. To investigate this further, we combined the police presence and police engagement conditions to explore the overall association in admitted drug use with and without a police chaperone (hypothesis 1c). As expected, more people admitted to taking drugs when police were not present (8%, *n* = 156), *χ*^2^(*df* = 1, *n* = 4722) = 38.74, *p* < 0.001; *φ* = 0.091. This represented a small effect in the association between the two variables, as reflected by less than 1% shared variance. However, another way of looking at this is through an odds ratio. The odds of a person admitting to taking drugs are 2.21 (95% CI: 1.71, 2.84) times greater when there was no police presence. Taken together, these results support hypothesis 1.

Those who admitted to drug use with the police present in any form were slightly older (x̅ = 22.71 years, *σ* = 4.5 years, *n* = 104) than those who admitted drug use without any police presence (x̅ = 21.47 years, *σ* = 3.8 years, *n* = 155). With such a small difference in age, we conducted an equivalence test based upon a five-year difference (as this age range difference is what is recorded by the Australian Bureau of Statistics as separate categories for alcohol use; Devilly and Kavanagh [15]). Based on Schuirmann’s two one-sided *t* test [39], we found this difference to be trivial (*df* = 257, *t*_upper_ − 7.245 > − 1.65, *t*_lower_− 12.02 > 1.65). There was also no difference between police presence versus no police presence on gender χ^2^(*df* = 1, *n* = 261) = 2.39, *p* = 0.12; *φ* = 0.096. There was also no difference between the conditions on whether they admitted multiple or single drug use, χ^2^(*df* = 1, *n* = 244) = 0.10, *p* = 0.75; *φ* = 0.02.

In effect, while there were fewer people who admitted to drug preloading in the police presence condition, we could find no demographic differences between those who admitted to drug preloading with and without a police presence. Therefore, we will combine the groups of police versus no police presence in our subsequent analyses to increase power when looking at the nature of drug preloaders. In summary, 8% of people who were asked without a police presence (and 5.53% of the entire sample) admitted to having preloaded with drugs before entering the NED. Obviously, we believe the number of actual drug preloaders to be much higher, but we are analyzing here the subsample of people who admit to drug preloading.

### Ancillary analysis—police presence

To investigate the dynamic between drug users and the police further, we assessed how approachable they believed the police to be in the area and whether they have used/required (but none was available) police assistance in the past. Most drug users (67.47%, *n* = 21) and non-drug users (75.37%, *n* = 477) rated the police in the area as approachable, with no proportional difference between groups, *χ*^2^(*df* = 1, *n* = 636) = 0.092, *p* = 0.334; *φ* = − 0.038. We did find that there was a statistically significant association between using drug use and having used police assistance (*χ*^2^[*df* = 1, *n* = 2362] = 7.78, *p* = 0.005; *φ* = 0.057) or requiring it when none was available (*χ*^2^[*df* = 1, *n* = 2362] = 4.85, *p* = 0.028; *φ* = 0.045). In both instances, the strength of the association was small, as reflected by less than 1% shared variance.

### Who preloads with drugs?

In support of hypothesis 2a, those who admitted using drugs, irrespective of police presence (x̅ = 21.96 years, *σ* = 4.13 years, *n* = 259), were significantly younger than non-drug admitters (x̅ = 22.74 years, *σ* = 6.27 years, *n* = 4448; *t* (*df* = 4705) = 1.96, *p* = 0.05). In a follow-up analysis examining the age differences between single- and poly-drug users, we found that there was not a statistically significant difference in age. However, poly-drug users (x̅ = 20.89 years, *σ* = 3.06 years, *n* = 44) were, on average, younger than single-drug users (x̅ = 22.17 years, σ = 4.16 years, *n* = 198; *t* (*df* = 240) = − 1.85, *p* = 0.07, Hedges’ *g* = 0.32). While this falls outside an alpha of 0.05, it does represent a probable small-to-moderate effect size. Looking at hypothesis 2b, there was a significant association between gender and admitted drug use, with males reporting a higher incidence than females, *χ*^2^(*df* = 1, *n* = 4723) = 21.38, *p* < 0.001; *φ* = 0.067. This was consistent across each of the drug types (except for hallucinogens) and practices listed. Overall, these results support hypothesis 2.

### Why do people preload with drugs?

Most people that admitted to preloading with drugs reportedly did so because they enjoyed the feeling (*n* = 27). Twenty-four said it was to save money, followed by to cope (*n* = 23), to get as high as possible (*n* = 22), and to socialize (*n* = 18). Eleven people said they preloaded with drugs for another reason and fourteen selected multiple reasons. Only two people from the entire sample reported using drugs because they were pressured into it from friends. It was also found that the reasons people gave for preloading with drugs did not have a significant influence on their entry BrAC scores, *F*(*df* = 6, 117) = 1.044, *p* = 0.401. In summary, hypothesis 3 (that drug preloading will predominantly be socially driven) was not supported.

### What drugs are people preloading with?

In support of hypothesis 4a, we found that people preloaded with a diverse range of drugs. As shown in Table 1, MDMA was the most used drug, followed by cannabis, and other stimulants (i.e., cocaine and amphetamines). A smaller proportion of admitted users preloaded with sedatives, hallucinogens, and heroin. A subset of the sample chose to not specify the type of drug used or selected ‘*other*,’ indicating the drug was not listed on the survey or the participant did not know what substance/s they had consumed. Looking at hypothesis 4b, we found that admitted drug users were more likely to have only preloaded with one drug (single-drug use = 76.63%, *n* = 200) than multiple drugs (poly-drug use = 16.83, *n* = 44). MDMA (70.45%, *n* = 31) and cannabis (59.09%, *n* = 26) were the two most used substances by poly-drug users. The combined use of other drugs occurred less frequently. Collectively, these results support hypothesis 4.

**Table 1:** Incidence rates, personal characteristics, and level of BrAC intoxication among self-disclosed drug preloaders

	Incidence	Age		Gender		BrAC reading	
	% (N)	$\overline{x}$(SD; N)	Range	Male % (N)	Female % (N)	≥ .000 $\overline{x}$ (SD; Median; N)	≥ .001 $\overline{x}$ (SD; Median; N)
*Drug used*							
MDMA	45.21 (118)	21.49 (4.00; 117)	18–41	65.25 (77)	34.75 (41)	.075 (.053; .067; 118)	.083 (.049; .080; 106)
Cannabis	38.31 (100)	21.44 (3.72; 100)	18–33	62.00 (62)	38.00 (38)	.065 (.054; .052; 100)	.075 (.051; .065; 87)
Cocaine	11.49 (30)	22.73 (3.93; 30)	18–33	76.67 (23)	23.33 (7)	.099 (.058; .105; 30)	.110 (.050; .109; 37)
Amphetamines	6.90 (18)	21.76 (3.46; 17)	19–29	77.78 (14)	22.22 (4)	.045 (.045; .039; 18)	.068 (.038; .062; 12)
Hallucinogens	2.30 (6)	22.67 (5.09; 6)	18–29	50.00 (3)	50.00 (3)	.037 (.046; .027; 6)	.074 (.036; .053; 3)
Heroin	2.30 (6)	21.83 (4.88; 6)	18–29	100.00 (6)	0.00 (0)	.059 (.051; .052; 6)	.059 (.051; .052; 6)
Sedatives	2.30 (6)	22.33 (3.33; 6)	20–29	83.33 (5)	16.67 (1)	.047 (.043; .033; 6)	.057 (.041; .043; 5)
Other	9.20 (24)	21.46 (3.24; 24)	18–31	62.50 (15)	37.50 (9)	.081 (.065; .068; 24)	.097 (.059; .104; 20)
Did not specify	6.51 (17)	23.00 (7.70; 17)	18–37	76.47 (13)	23.53 (4)	.084 (.037; .075; 17)	.084 (.037; .075; 17)
*Method of use*							
Single-drug user	76.63 (200)	22.12 (4.16; 198)	18–41	65.50 (131)	34.50 (69)	.075 (.056; .065; 200)	.085 (.052; .080; 177)
Multi-drug user	16.86 (44)	20.89 (3.06; 44)	18–29	63.64 (28)	36.36 (16)	.066 (.054; .056; 44)	.083 (.048; .083; 35)
*Drug use overall*							
Yes	5.53 (261)	21.97 (4.13; 259)	18–41	65.90 (172)	34.10 (89)	.074 (.055; .065; 261)	.084 (.050; .080; 229)
No	94.47 (4462)	22.74 (6.27; 4448)	16–64	51.19 (2284)	48.81 (2178)	.055 (.051; .047; 4462)	.075 (.047; .068; 3288)
Total	100.00 (4723)	22.70 (6.18; 4707)	16–64	52.00 (2456)	48.00 (2267)	.056 (.052; .048; 4723)	.076 (.047; .069; 3517)

### For those preloading with drugs, how affected do they feel?

In line with hypothesis 5, we find that those who admitted drug use were more likely to also have a BrAC above zero (87.74%, *n* = 229) than to have a zero BrAC (12.26%, *n* = 32). However, this represented a smaller percentage of ‘zeros’ compared to non-drug users where BrAC above zero represented 72.69% (*n* = 3288) and zero readings represented 26.13% (*n* = 1174) of their sample. This was highly significant but represented only 0.55% of explained variance (*χ*^2^ (*df* = 1, *n* = 4723) = 25.60, *p* < 0.0001, φ = 0.074).

Further, people who admitted to drug use were significantly more inebriated (x̅ = 0.074, *σ* = 0.055, *n* = 261) than non-drug users (x̅ = 0.055, *σ* = 0.052, *n* = 4462), with a small-to-moderate effect size (Hedges’ *g* = 0.36). When we removed people who blew zero on alcohol (irrespective of admitted drug use), this effect size dropped (Hedges’ *g* = 0.19). However, this was still significant with drug users (x̅ = 0.084, *σ* = 0.05, *n* = 229) blowing higher on alcohol intoxication than those who did not admit to drug preloading (x̅ = 0.075, *σ* = 0.047, *n* = 3288). Overall, this supports hypotheses 5a and 5b.

**Fig. 2:**
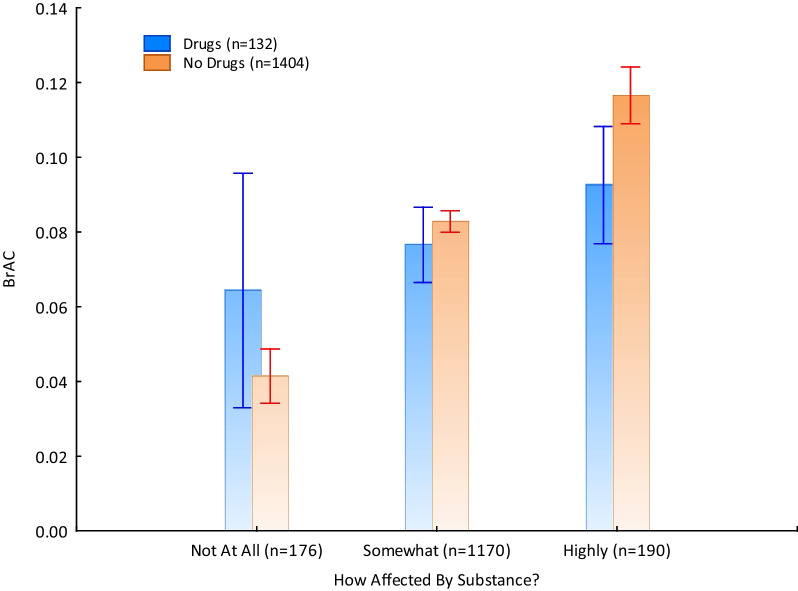
A comparison of subjective and objective markers of inebriation between drug users and non-drug users

Looking at how affected people reported to be by substances, we first needed to create a variable for inclusion for people who either said ‘*yes*’ to drug use and for those who, if they said ‘*no*’ to drug use, had a BrAC over zero. Otherwise, we would be creating a strawman argument, comparing one group who had ingested an intoxicating substance (i.e., drugs) being compared to some people in the other group who hadn’t (i.e., no drugs and no alcohol). Conducting an analysis of variance (ANOVA) test with one dependent variable (BrAC reading) and two independent variables (drug use: ‘*yes*’ and ‘*no*’; affected by substances*:* ‘*not at all*,’ ‘*somewhat*,’ and ‘*highly*’), we found no significant main effect for drug use (*F*[*df* = 1, 1530] = 0.15, *p* = 0.70) but, as one would expect, an effect for how affected people felt in relation to their BrAC (*F*[*df* = 2, 1530] = 19.17, *p* < 0.0001). In line with hypothesis 5c, there was a significant interaction effect between drug use and how affected people reported to be by substances on BrAC (*F*[*df* = 2, 1530] = 3.45, *p* = 0.03). As can be seen in Fig. 2, people who reported feeling not at all affected, and reportedly only consumed alcohol, presented as less intoxicated than drug users. Those who used drugs and reported feeling not at all affected presented more intoxicated and showed greater variation in level of intoxication from preloading. In contrast to this, a reversed outcome was found for people who reported feeling somewhat and highly affected by their preloading intoxication.

## Discussion

The key objectives of this research were to (1) investigate the nature of drug preloaders as they entered the NEDs and (2) see whether police presence greatly affected our results. Overall, we found that there was a significant difference in admitted drug use between there being a police presence and no police presence (collectively supporting H1a and H1c). Although there was not a significant difference between the police presence and engagement conditions (unsupportive of H1b), there was an increased trend in disclosed drug use after people had a positive interaction with police officers. Our results also revealed that people who admittedly preloaded with drugs were: younger than non-drug users (H2a); more likely to be male than female (H2b); motivated to preload with drugs because they enjoy the feeling drugs provide rather than to socialize (unsupportive of H3); likely to preload using variety of different drug types (H4a); more likely to be a single-drug user than a poly-drug user (H4b); more likely to be a polysubstance user than a drug-only user (H5a); significantly more intoxicated than non-drug users (H5b); and reported feeling more affected by their preloading of substances than non-drug users, but not as BrAC intoxication increased (partially supportive of H5c).

### The implications of the police presence

In line with conventional standings, collecting sensitive data from admitted drug users was best done in the absence of police. Although there was a significant difference in reporting between the groups, people were remarkably willing (very little difference) when police were around or not. We put this down to our presence being a known component in the NED. We were also from a university, and even knowing the police were present, we pointed out that they were there for our safety and would keep the results confidential. While we found there to be no meaningful difference of admitted drug use between the presence and engagement conditions, the disclosure rate was slightly higher after the participant shared a positive interaction with a police officer. This presents an interesting finding when taking into consideration that the effect of having a police presence verse no police presence was small. To examine this further, we looked at how approachable people felt the police were within the NED and found that ratings did not differ between self-disclosed drug users and non-users. However, it was revealed that admitted drug users were more likely to have used and/or require police assistance on past visits to the NED.

From a methodological standpoint, we can infer that we assessed a subset of drug users who were willing to participate due to their past experiences. While for some (i.e., those who required assistance, but none was available) this experience may have had negative consequences, their participation in the study (irrespective of whether they admitted to using drugs during the current session or not) suggests that there are still perceived benefits around engaging with the police. In essence, it would be interesting to know what proportion of drug users intentionally denied using drugs on the survey versus those who declined to participate all together. Although this subset of drug users was not forthcoming around disclosing this information, they were trusting enough to participate in the research/intervention. Collectively, these findings highlight the practical utility of having a friendly police presence operating within this context, particularly when this presence was paired with a positive community service, such as providing and interpreting BrAC tests. From our observation and discussion with participants, this exchange appeared to be an adaptive way for the police to connect and establish rapport with a particularly vulnerable subset of the population. We speculate that fostering this relationship will lead to a positive reduction in harm within this context.

### The nature of drug preloading

In line with Miller, Voas, and colleagues (North American research team) and Sanchez and colleagues (South America research team), we too found most people were inebriated at the point of assessment (c.f. [6, 19, 28, 28, 29, 29, 32, 33, 38]. To contrast our results (obtained using a point-of-entry design) with trends identified in their results (obtained using a portal-in design), we found that alcohol was the most used substance during the preloading phase, with a much lower proportion of people disclosing having used drugs. However, the prevalence rate of reported drug use was much lower in the present study than the studies conducted out of North and South America. This result is likely due to various methodological differences. In our methodology, for instance, we periodically collected data in the presence of police, we relied on self-report data, and we used a point-of-entry design. Conversely, the North and South American research groups did not disclose having a police presence and they likely obtained a more accurate representation of drug use as they used objective measures. However, one cannot attribute the entire incidence rate of drug use to preloading as both research teams used the portal-in approach. This would inflate the estimates of preloading because people may have consumed substances in other bars and clubs prior to participation. In line with this, estimates of intoxication severity could have been deflated in studies that excluded people based on how inebriated a researcher perceived them to be (thereby underestimating the full breadth and severity of preloading intoxication in that area). This difference could also stem from various geographical (e.g., affordability, accessibility, and differences in laws surrounding the use of tobacco and other drugs) and cultural (e.g., stigma and social acceptability) factors.

Consistent with Miller et al. [28, 29], we too found that males were more likely to admit having preloaded with drugs than females. This finding was consistent across most drugs and practices (i.e., single- and poly-drug use). Exploring this further, we found that most people in our study who drug preloaded did so with only one type of drug, while other researchers report their sample being predominantly polysubstance users [28, 29]. In line with past research (i.e., [6, 19, 28, 28, 29, 29, 32, 33, 38]), we too found a high incidence rate of cannabis use among drug users. This makes sense given to the impracticalities of using cannabis after having transitioned into Australian NEDs. In contrast to these studies, it was revealed that more people opted to preload with MDMA (and to a lesser extent other stimulants) than other types of drugs (which were used far less often). The reason for this could be due to legal and/or accessibility differences throughout each geographical location. For example, in 2018, the cost of a gram of cocaine in Australia was €205.1 per gram (AUD$302.75), compared to €56 (AUD$82.7) in the USA and €12.5 (AUD$18.46) in Brazil [43]. Another explanation for this difference could be that MDMA and/or other stimulants (particularly those in pill form) are much easier to conceal and covertly use than cannabis and cocaine in its powered form. Further, we did not check the drug, which was self-reported. It may be the case that the suspected MDMA may really be amphetamines of some variety considering the similarity in bodily response yet the difference in price. In effect, the use of MDMA and other stimulants could also provide an explanation for why drug users felt less effected from their substance use than non-drug users at lower inebriation rates. However, it took less alcohol for drug users to feel the effect of the interaction at higher inebriation levels. In effect, at lower inebriation rates drug users have a masking effect of the alcohol, but this quickly changes at higher levels. It is reasonable to assume that these people are at higher risk for harms as the night progresses.

In contrast to Miller et al. [33], Miller et al. [32], and Miller et al. [28, 29], we found drug preloaders to be younger in age than non-drug users. Younger drug users displayed a greater propensity to engaging in poly-drug use than older participants. When we consider this in conjunction with peoples’ motivations to drug preload, younger participants may preload with multiple drugs to reduce financial expenditure over the course of the night while also entering the NED in their desired state of inebriation. In turn, an area of risk for young people likely stems from their lack of experience and proper guidance around navigating their use of substances. Conversely, the preloading practices of older participants may reflect greater refinement and propensity to be able to afford more expensive drugs such as cocaine. Considering cocaine users were slightly older and presented with higher BrAC readings than non-cocaine users, an area of risk for older people who drug preload likely stems from the ‘masking effect’ stimulants have on alcohol. In effect, these finding align with Miller et al. [28, 29].

In Australia, the maximum BrAC for a driver to still operate a car is a BrAC concentration of 0.05% (which estimates that there is 0.05 g of alcohol in one deciliter of blood; [9]). In some of the USA, some of the UK and Canada, for example, the driving limit for alcohol is a BrAC of 0.08%. However, there is evidence that reducing the limit even from 0.05 to 0.03% significantly reduces crashes [11]. One can estimate from this that even at lower levels, small increases in BrAC have detrimental cognitive and/or physical effects. A reading of over 0.15% introduces the possibility of breathing and walking difficulties, and above 0.30% can lead to coma and death. Adding other drugs to alcohol can have unplanned consequences. In some cases, the acute alcohol intake may, paradoxically, reduce the effect of the drug, while the effect of the drug can be enhanced with chronic alcohol intake. With such unpredictable outcomes based upon different drugs, the interactional effects of alcohol and drugs represent a distinct risk of harm to the taker and people near to them.

### Strengths, limitations, and future directions

This study presents information unique in several manners. To the best of our knowledge, drug preloading was yet to be operationalized and explored in conjunction with alcohol preloading. In addition to being a multi-site field-based investigation, the point-of-entry design had also never been applied to determine the personal factors and behavioral nuances of drug preloading. Until now, the relationship between polysubstance preloading and entry level intoxication was also yet to be determined. In examining this, we used a real-world sample of systematically selected people and did not rely on a university student sample participating for course credit. Moreover, we also did not exclude people based on how inebriated we perceived them to be and interpreted BrAC readings on-site.

A key limitation in the present study was that we relied on people self-disclosing their use of drugs, which in turn may have contributed to the large imbalance of drug preloaders and non-drug users surveyed. Despite this imbalance, we find our prevalence estimates to be consistent with estimates (i.e., typically ≤ 10%) provided in similar field research (e.g., [8, 14, 15, 27, 30, 31, 35, 36]). To obtain more accurate estimates, collecting biological samples (e.g., blood, urine, sweat, or oral fluid) has become a popular alternative, with estimates ranging from 14 to 45% (e.g., [4, 5, 24, 27, 30, 31, 33]). In effect, this could be seen as a protective factor for approximately 10% of people who self-disclose drug use in the vicinity. Comparing this figure to those using confirmatory tests, one way of looking at this is that approximately a quarter to a half of drug users are prepared to disclose having consumed drugs on a given night out. Another way of looking at this is that approximately 10% (and more) of this population are placing themselves at greater risk of harm than non-drug users. Considering the large number of people that occupy this space, a minimum approximation of 10% (with this figure likely to be higher) of people using drugs still reflects a large number on any given night out. While we agree that confirmatory drug testing inside NEDs (and festivals) for research and substantive purposes is important, this approach has a myriad of problems. For example, some methods are invasive (e.g., urine or blood testing), certain drugs can make it difficult to produce saliva, obtaining biological markers could elicit suspicion, it can be time-consuming (which has shown to be a key reason people refuse to participate), and it can be expensive. Besides using confirmatory tests and/or taking people at their word, an alternative approach could be to use screening equipment (e.g., ion mobility spectrometry). This will be looked at in future research.

Although we acknowledge the design imbalance (as well as the age of the data) as limiting factors, we contend that the following should also be acknowledged: (a) Drug use has shown to be common and extensive among this population; (b) both drug use and preloading, respectively, are strong predictors of harm; (c) there is a paucity of research linking these two concepts together (this being the first study seeking to fully understand this phenomenon specifically in this context); (d) preloading-related concerns only appear to be worsening (i.e., there is an increasing trend in people engaging in this practice; people are entering NEDs later, they are more intoxicated when they arrive, and now it has been demonstrated that people are also preloading with drugs as well as alcohol) since the introduction of legislative change in Queensland, Australia [14, 15]; and (e) the challenges associated with the collection of drug-related data in field research are well documented, so transparently sharing ones’ successes and failures is pertinent to minimizing potential sources of error in epidemiological research [23]. With this in mind, we posit that any information is better than no information where there is an element of risk involved. From an ethical perspective, now knowing full well that there is a particularly vulnerable subset of the population susceptible to experiencing harm in NEDs, we encourage researchers to broaden the scope with which they conceptualize preloading. To derive meaningful solutions to a problem, it first needs to be fully understood. If we continue to focus exclusively on alcohol preloading, it is apparent that will overlook a predominant factor exacerbating and perpetuating harm. Only then can we look at interventions to reduce harm. For example, interventions looking at the interaction of alcohol and other drugs could be targeted at a population level through advertising. Sub-population interventions could even occur within the NEDs or inside the actual clubs. It has been our experience that patrons frequently have no concept of BrAC levels and providing these breathalyzers may themselves reduce harm (cf. [13]. Likewise, pill testing or (in the case of unfavorable legislation) ion trap mobility spectrometry swabbing of people may have a large impact on overdoses and general toxicity, particularly when combined with alcohol.

Future research should also consider applying the same methodological principals of the portal (in and out) design to the broader event-level session. For instance, rather than as they portal-in and portal-out of a specific nightclub, this could be achieved by assessing people at points of entry and points of exit to the NED. The utility of this is that it would provide greater sensitivity to the collection of preloading data as well as the target-event, as opposed to a single venue or event. Consistent with this, to obtain accurate estimates from people who preload with drugs (and alcohol) using a within subjects’ design would be of great benefit to furthering our understanding of this practice and the harms experienced by people who drug preload over the course of a night out. Considering other practical uses of this methodology, engaging with people as they enter and exit NEDs could prove to be valuable intervention points. From our observation and discussion with participants, we found providing free BrAC tests proved to be a simple and effective intervention in and of itself. While completing the survey questionnaire, we found people displayed genuine interest and curiosity about BrAC (and drug) assessment. After participation had finalized, we found this provided space for our research team and/or police officers to educate participants on their level of intoxication and offer suggestions (e.g., to slow down the pace of consuming alcohol or to visit a rest and recovery space) to people. At exit, the research team would be more likely encourage people to use public transport and connect highly intoxicated people with police or other street-based services.

## Conclusion

Throughout this investigation, we sought to bridge the gap between alcohol preloading and drug use by examining this phenomenon under various levels of police presence. In doing so, we used a point-of-entry design to address some of the conceptual, ethical, and methodological challenges field-based researchers’ experience. We found people who drug preload to be a particularly vulnerable subset of the population. We found them to be younger in age than non-drug users, more likely to be male than female, use one type of drug (mostly stimulants) rather than multiple if we exclude alcohol), polysubstance users when we include alcohol, significantly more intoxicated upon arrival, and less affected from their substance use (but only as BrAC levels increased). Although having a police presence was found to have a statistically negative impact on data collection, sharing a positive interaction with an officer could be regarded as a positive intervention in and of itself—particularly for younger partygoers. Given the paucity of research on the topic of drug preloading, further enquiry is needed to better understand the nature of this practice and the harms associated with it. We believe that the first step in this quest is to obtain a quick, reliable, cheap, objective test of the drugs reportedly being used in the NEDs to clarify the current research and needed interventions.

## Supplementary Information

Additional file 1.

## Footnotes

1 Drug use here refers to the consumption of illicit substances (i.e., the substance was manufactured, distributed, and/or consumed for recreational purposes and not to treat a medical condition under the supervision of health care professional).

2 This policy introduced several strategies that restricted the sale and service of alcohol toward the end of the night, ceased off-sale trading licenses at 10 p.m., and, later, introduced ID Scanning.
