# Supplementary material for: Preloading with drugs before entry to the nighttime entertainment district: presentation, intoxication rates, and effects of police presence during assessment
Source: Harm Reduct J. 2023 May 2;20:62. doi: 10.1186/s12954-023-00749-2 (PMC10152730; doi:10.1186/s12954-023-00749-2)
Supplement: Supplementary file 1 — Additional file 1. [file 12954_2023_749_MOESM1_ESM.pdf]

Supplementary Information 1

Ethical considerations

In the earlier stages of this project (prior to the legislation change), a police chaperone accompanied researchers during data collection with the aim of providing a safe and positive presence in the NED. At this time, officers maintained a presence around the vicinity of data collection but did not actively engage with participants. This created a dilemma whereby we wanted to obtain reliable and accurate estimates of pre-loading intoxication without excluding those who are heavily intoxicated and/or were suspected drug-users but were bound by the initial ethical constraints of having a police chaperone to ensure the safety of all involved. Conventional wisdom would suggest that most people would elect not to disclose their drug use around police out fear there would be legal ramifications should this be brought to light. The implication of this led us to having less confidence when reporting on the behaviors of drug-users. Overtime, we became known as a positive presence in the NED by both patrons and NED workers and were able to develop a greater awareness of the risks associated with collecting data in the NED environment. After the legislation change it became apparent that the police chaperone did not affect the type of participants we engaged and was not required as a means of security for researchers (Devilly, 2018). Recognizing this and having demonstrated a precedence of collecting data over a lengthy period, we received approval by our ethical review board to progress our methodological approach with the absence of police. From this, we sought to determine what impact varying levels of police exposure has on peoples' willingness to admit to having used drugs in this context. To explore this further in the present study, we create another condition and include participants who actively engage with the police chaperone.

On occasion, participants would enquire as to whether researchers were police in disguise and/or what their relationship was with the officers. In these instances, researchers were transparent with participants in their role, what their relationship with the police officers was, and in explaining that all information collected would remain private and confidential. To mitigate confusion researchers wore black University polo shirts (distinct from a Police Officer uniform) and displayed their staff / student identification in clear view for participants to observe. As a positive community service, we offered those who refused or were ineligible to participate (i.e., patrons who had already transitioned into the

NED) a free BrAC test and recorded no data. Refusals were mainly due to people being in a hurry or being with people who did not want to wait around while they participated. To date, no participant has contacted us requesting their data be excluded and destroyed.

Supplementary Information 2

Survey

Data collected from the entry questionnaire pertained to participant:

i. Demographics: *How old are you? What is your gender?;*

ii. Drug pre-loading practices: *Did you have any party drugs before coming out tonight? If so, what party drugs have you had tonight?* (response options included: '*amphetamines*', '*benzodiazepine*', '*cannabis*', '*cocaine*', '*hallucinogens*', '*heroin*', '*MDMA*', '*sedatives*', and '*drugs other than those specified*');

iii. Motivation: *What was your reason for pre-loading with party drugs?* (response options included: '*save money*', '*socialise*', '*get as high as possible*', '*to cope ['feel more relaxed or increase confidence*', '*enjoy the feeling*', '*peer pressure*', or they did so for '*multiples reasons*');

iv. Perceived intoxication level: *How affected by drugs and / or alcohol do you feel?* (response options ranged on a five-point Likert scale: '*not at all affected*', '*a little affected*', '*somewhat affected*', '*highly affected*', and '*very highly affected*'); and

v. Level of intoxication (Blood Alcohol Concentration level or approximated via breath testing).
